# Muscular activities during sling- and ground-based push-up exercise

**DOI:** 10.1186/1756-0500-7-192

**Published:** 2014-03-28

**Authors:** Sumiaki Maeo, Tatsuya Chou, Masayoshi Yamamoto, Hiroaki Kanehisa

**Affiliations:** 1 National Institute of Fitness and Sports in Kanoya, 1 Shiromizu, Kanoya 891-2393, Japan; 2 Research Fellow of the Japan Society for the Promotion of Science, 5-3-1 Kouji, Tokyo 102-0083, Japan

**Keywords:** Instability, Co-contraction, Electromyography

## Abstract

**Background:**

This study aimed to clarify the characteristics of muscle activities during push-up exercises performed under sling condition by comparison with those performed under ground condition. We hypothesized that sling-based push-ups induce higher muscle activities than the ground-based push-ups, and its effects are more prominent in dynamic compared to static exercise owing to increased demands of stabilization.

**Findings:**

Twenty young males performed sling- and ground-based push-ups in each of static (maintaining the posture with the elbow joint angle at 90 deg) and dynamic (repeating push-ups at a rate of 45 per minute) exercises. Surface electromyograms (EMGs) of the pectoralis major, latissimus dorsi, triceps brachii, biceps brachii, rectus abdominis, external oblique, internal oblique, and erector spinae muscles were recorded during the exercises. The EMG data were normalized to those obtained during maximal voluntary contraction of each muscle (% EMGmax). In the static exercise, sling condition showed significantly higher % EMGmax values than the ground condition in the triceps brachii (+27%: relative to ground condition) and biceps brachii (+128%) as well as the three abdominal muscles (+15% to +27%). In the dynamic exercise, such condition-related differences were more prominent and those in the pectoralis major (+29%) in addition to the aforementioned five muscles (+19% to +144%) were significant.

**Conclusion:**

These results supported the hypothesis and indicate that sling-based push-up exercise can provide greater activation in upper limb and anterior trunk muscles than the ground-based push-up exercise.

## Findings

### Introduction

Instability resistance training devices are very popular in current training facilities, and the use of such devices to train the trunk musculature is an essential feature of many training facilities and programs [1]. Instability resistance training involves exercises either with body mass as a resistance or external loads (e.g. dumbbells, barbells) that are performed on an unstable surface or using unstable devices [2]. In the past, these types of exercises have been applied to only individuals with low back problems in physical therapy clinics [3]. In recent years, however, fitness professionals have increasingly emphasized trunk stability exercises in sports conditioning programs, and it is now considered that greater trunk stability may benefit sports performance by providing a foundation for greater force production in the upper and lower extremities [4] although there is not enough evidence to establish a clear relation between the practice of these exercises and the improvement in sports performance [5].

Recently, sling-based exercise has been involved in both athletic and fitness training programs [6-8]. Sling-based exercise is a novel form of exercise that allows an individual to use his or her body weight (in suspended condition) to provide resistance [7]. Several studies have demonstrated that training interventions that used sling-based trunk exercises produced significant performance improvements in both untrained and trained individuals [6,8,9]. For example, Dannelly et al. [6] reported that sling-based exercises were as effective as traditional resistance training for strength gain during the initial phases of a strength training program in untrained females. Prokopy et al. [8] and Saeterbakken et al. [9] showed that sling-based training, but not traditional resistance training, increased throwing velocity in female athletes. These findings support the efficacy of sling-based exercise for improving muscle function.

On the other hand, little information concerning muscular activities during sling exercise is available from the literature. To our knowledge, only one study [10] examined the muscular activities during sling-based exercise and compared them with those during ground-based exercise. In their study [10], it was shown that sling-based push-ups caused higher activation in some of the trunk muscles than traditional ground-based push-ups. However, they mainly focused on the activities of abdominal and lower back muscles. The influence of using sling on the activities of other important muscles for performing push-ups, such as pectoralis major and triceps brachii muscles [11,12], is unknown. Also, they used only dynamic push-ups for comparison of muscle activation between sling and ground conditions. Because a training program usually consists of different types of movement pattern [13,14], for example static and dynamic exercises, the influence of such difference in exercise types on muscle activities should also be clarified. In fact, as mentioned above, sling-based exercise is now performed by a wide range of population, and it is often considered that static exercises are recommended for inexperienced individuals or patients with lower back pain while dynamic exercises involving multi-joint movements are advisable for advanced trained individuals [13-15].

In this study, we aimed to clarify the characteristics of muscle activities during sling-based exercise with respect to its difference from ground-based exercise. Push-up exercise was chosen because this exercise was performed in all of the previous studies that involved sling-based resistance training [6,8,9], and thus it can be considered as the representative sling exercise. We hypothesized that sling-based push-ups induce higher muscle activities than the ground-based push-ups, and its effects are more prominent in dynamic compared to static exercise owing to increased demands of stabilization.

### Methods

#### Subjects

Twenty male university students majoring in physical education participated in this study. The means and SDs of their age, body height, and body mass were 21.4 ± 2.3 yrs, 167.6 ± 5.2 cm, and 62.5 ± 6.9 kg, respectively. All subjects were physically active, and were well familiarized with performing both sling- and ground-based push-ups in both static and dynamic exercises. This study was approved by the Ethics Committee of the National Institute of Fitness and Sports in Kanoya and was consistent with institutional ethical requirements for human experimentation in accordance with the Declaration of Helsinki. Prior to the measurement session, all subjects were fully informed about the procedures and possible risks involved as well as the purpose of the study, and their written informed consent was obtained.

#### Procedure

Isometric maximal voluntary contractions (MVCs) for each muscle were performed for the purpose of normalization. Force during isometric MVC was measured using a custom-made force-measurement device with a tension/compression load cell (LUR-A-SA1; Kyowa, Japan). The force signal obtained via a 16-bit A/D converter (Power Lab 16 s; ADInstruments, Australia) was recorded on a personal computer at a sampling frequency of 2,000 Hz. In the MVC tasks, as well as subsequent push-up tasks, the surface electromyograms (EMGs) of the trunk and upper extremity muscles were recorded. After warming-up and a rest period of 3 min, the subjects were encouraged to exert maximal force (progressively increasing the force taking about 5 s) two times with at least 3 min between trials to exclude the influence of fatigue. Additional trials were performed if the difference in the peak forces of the two MVCs was greater than 5%. The trial with the highest peak force was selected for analysis. None of the subjects reported pain and/or discomfort during all MVCs, and we made sure that all subjects could perform the tasks with maximal effort. The postures and tasks of MVCs were determined in a pilot study where various MVC techniques were performed and those which maximized the force and EMG activity of target muscle were selected. Each MVC was performed as follows.

##### Chest press

**Figure 1:**
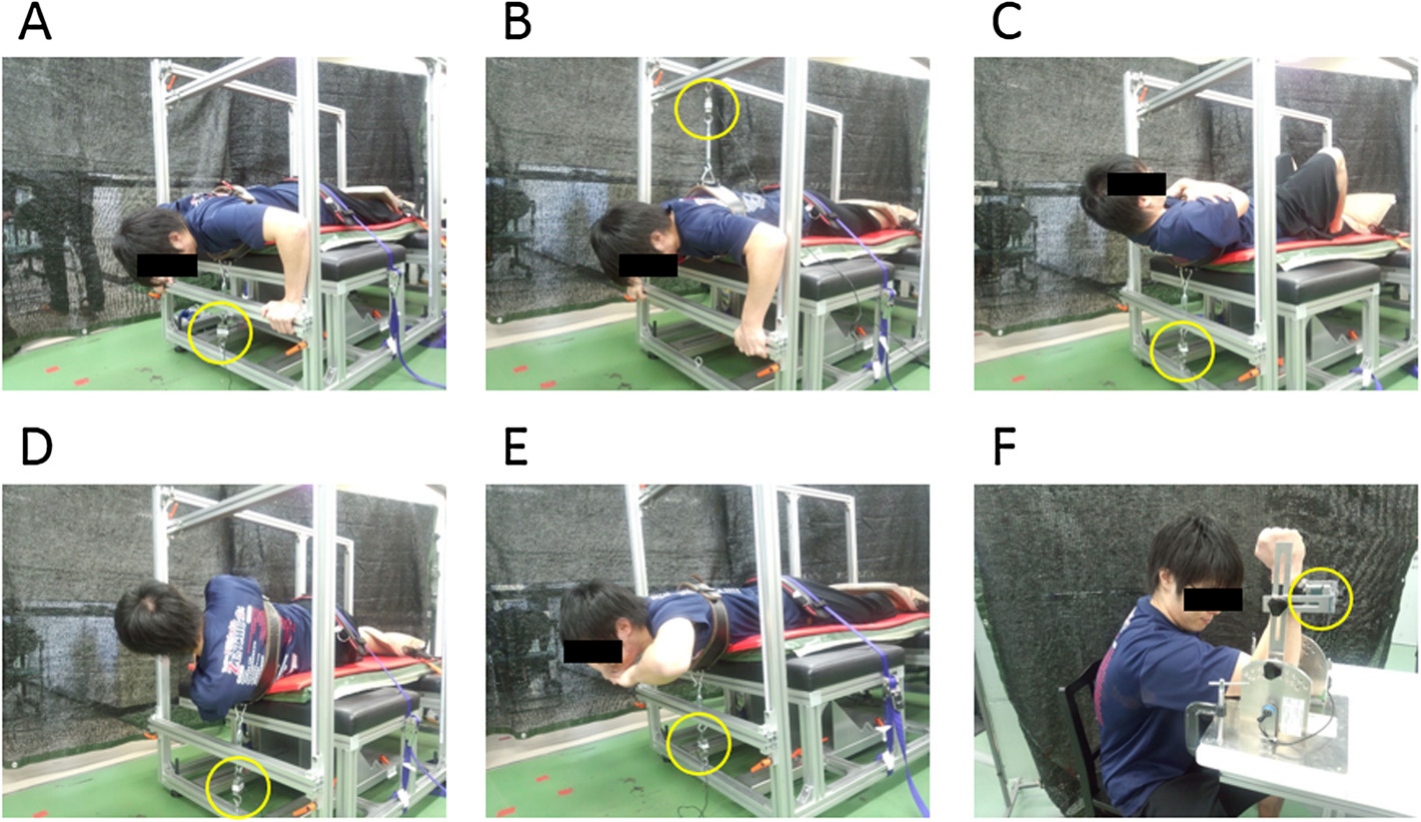
**Pictures of MVCs; chest press (A), prone row (B), trunk flexion (C), lateral trunk flexion (D), trunk extension (E), and elbow flexion/extension (F).** Yellow circle indicates the location of load cells. Informed consent for the use of the pictures was obtained.

The subjects lay prone on a stable bench with the legs extended and the hips and feet fixed on the seat with a strap. By the use of a custom-made belt linked with a chain, which covered the upper torso and was securely connected to the load cell, the subjects were held tightly in position. The position of the belt was adjusted for each subject so that the belt covered the chest of the subjects. With the shoulders abducted at 90 deg and elbows flexed at 90 deg, the subjects performed isometric chest press (Figure 1A).

##### Prone row

In the same position as for the chest press, the subjects performed isometric prone row (Figure 1B).

##### Trunk flexion

The subjects lay supine on the seat with the knees flexed, the feet flat on the seat and fixed with a strap, and the upper torso connected to the load cell using the belt. The subjects then performed isometric trunk flexion (Figure 1C).

##### Trunk lateral flexion

The subjects lay on their left side on the seat with the legs extended, the hips and feet fixed on the seat with a strap, and the upper torso connected to the load cell using the belt. The subjects then performed isometric lateral flexion (bending right) (Figure 1D).

##### Trunk extension

The subjects lay prone on the bench with the legs extended, the hips and feet fixed on the seat with a strap, and the upper trunk connected to the load cell using the belt. The subjects then performed isometric trunk extension (Figure 1E).

##### Elbow flexion/extension

The subjects sat upright with the upper arm horizontal to the ground, the elbow joint angle at 90 deg, the forearm in a neutral position and the wrist fixed to a strap linked to the load cell. The subjects then performed elbow flexion or extension (Figure 1F).

**Figure 2:**
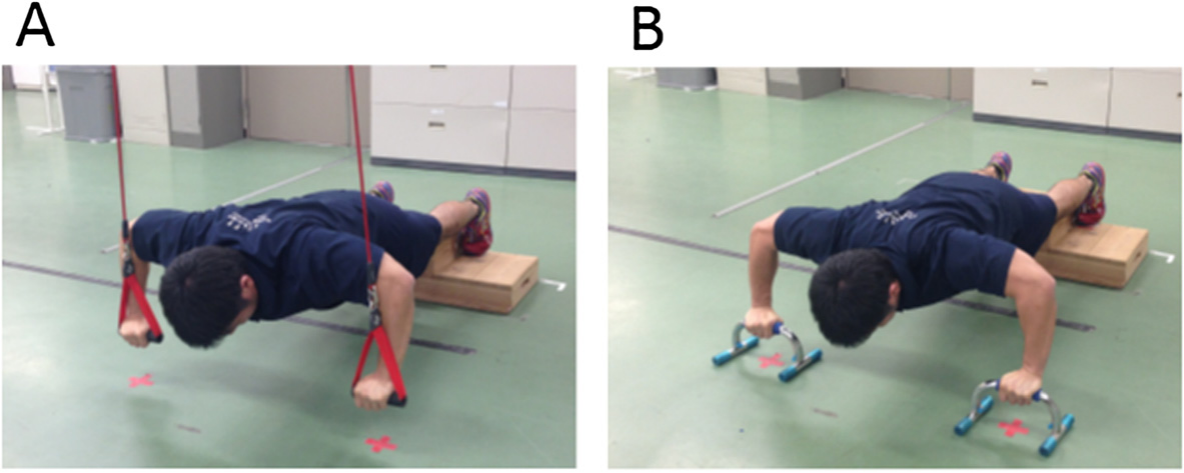
Pictures of static push-up exercise; sling (A) and ground (B) conditions.

**Figure 3:**
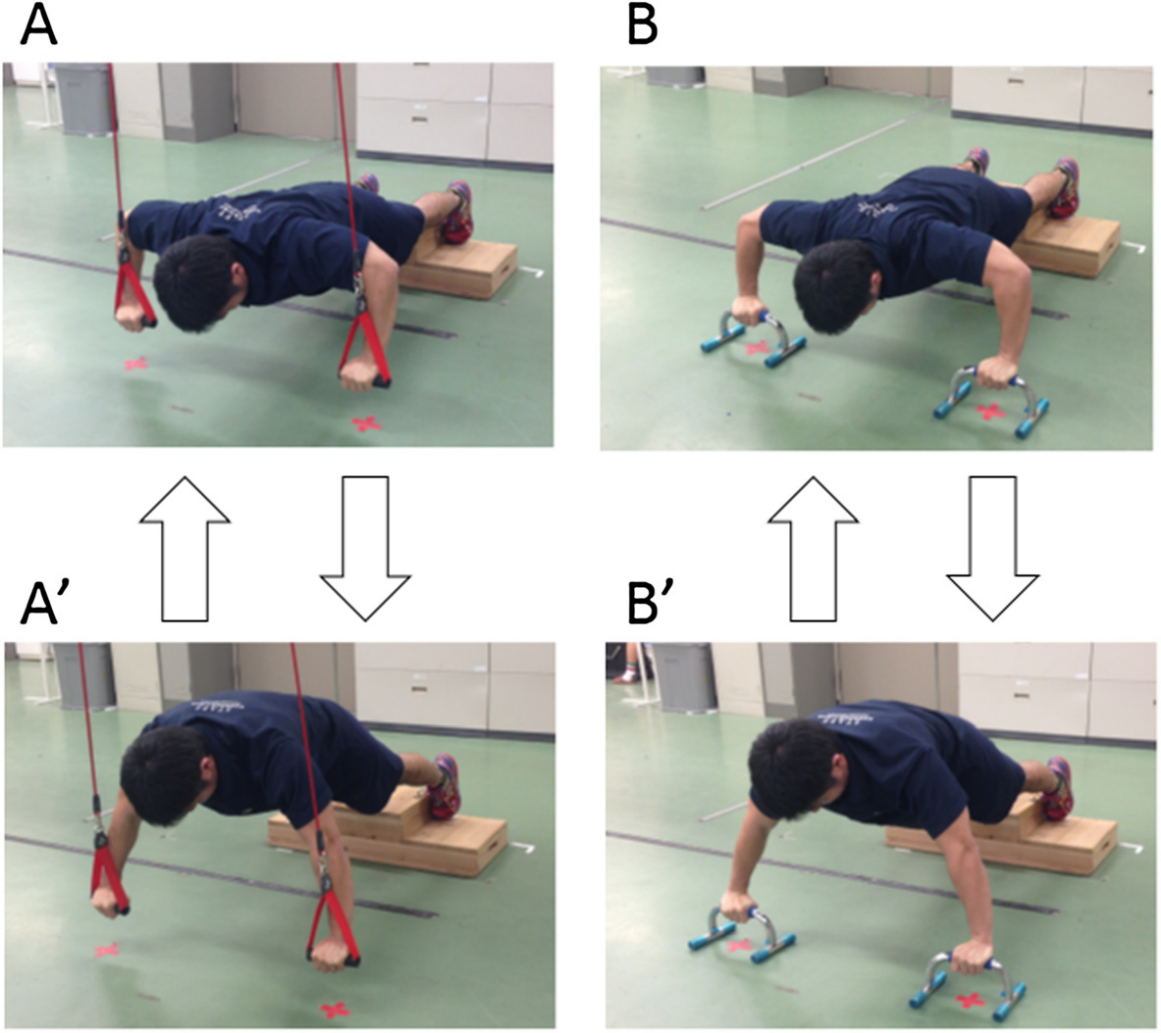
Pictures of dynamic push-up exercise; lowered (A and B) and raised (A’ and B’) phases of sling and ground conditions.

After the completion of MVC tasks, the subjects performed both sling- and ground-based push-ups in each of static and dynamic exercises in a random order across conditions and exercise types. For both sling and ground conditions, the shapes and heights of the grips were matched by using grip attachments and push-up bars for the sling and ground conditions, respectively (Figures 2 and 3). During the exercises, the elbow joint angles were measured using an electrogoniometer (SG110; Biometrics, UK), recorded together with the EMG activities, and stored in a personal computer. After the subjects were instructed and familiarized with the tasks, a measurement trial for each task was performed with at least a 3-min rest interval between each trial. Additional trials in each task were performed if either the subject or the researcher considered that the task performed was unsuccessful. Static exercise was maintained for 10 s and dynamic exercise was repeated 10 times at a rate of 45 per minute using a metronome, and each exercise was performed as follows.

##### Static exercise

In a prone position with the shoulders abducted at 90 deg, the elbows flexed at 90 deg, the hands grasping the grips, the pelvis raised off the floor, and the body weight distributed on the hands and toes, the subjects were instructed to maintain a flat position (Figure 2).

##### Dynamic exercise

In the same position as for the static exercise, the subjects were instructed to repeat push-up movements 10 times at a rate of 45 per minute (Figure 3).

#### EMG measurements and analysis

In the isometric MVC and push-up exercise tasks, the surface EMG activities of the pectoralis major (on an angle midway between the anterior aspect of the humeral head and the nipple over the muscle belly), latissimus dorsi (lateral to T9 spinous process over the muscle belly), triceps brachii long head (midway between the posterior crista of the acromion and the olecranon at 2 finger widths medial to the line), biceps brachii long head (midway between the anterior aspect of the humeral head and the elbow joint), rectus abdominis (3 cm lateral to the umbilicus), external oblique (~15 cm lateral to the umbilicus), internal oblique (1 cm medial to the anterior superior iliac spine), and erector spinae (3 cm lateral to L3 spinous process) muscles on the right side were measured by a bipolar configuration using a portable EMG recording apparatus (ME6000T16; MEGA Electronics, Finland). The electrode locations described by previous guidelines [16,17] were followed. Ag-AgCl electrodes of 15 mm diameter (N-00-S Blue sensor; Ambu, Denmark) were attached over the bellies of the muscles with an interelectrode distance of 20 mm after the skin surface was shaved, rubbed with sandpaper, and cleaned with alcohol. Another electrode for each muscle was attached and functioned as a ground electrode as well as a preamplifier. The EMG signals were 412-fold-amplified through the preamplifier, A/D-converted through a band-pass-filter (8–500 Hz/3 dB) at a sampling frequency of 2,000 Hz, and stored in a personal computer together with the elbow joint angle data for later analysis. From EMG data, the root-mean-square (RMS) amplitude of EMG for each muscle was calculated using data analysis software (Chart version 7; ADInstruments, Australia). In the MVC task, the maximum amplitude of EMG (EMGmax) in each muscle was determined over a 500-ms window centered on the time at which peak torque was attained [18,19]. For each muscle, the highest EMG amplitude obtained during all MVC tasks was adopted as EMGmax. The EMGs of each muscle during push-up exercise tasks are expressed as the value relative to the maximum (% EMGmax) [18,19]. EMGs during static exercise were analyzed as a mean value in an 8-s window following the first 1 s after steady contractions were achieved. EMGs during dynamic exercise were analyzed as a mean value during the 2^nd^ – 9^th^ repetitions based on the elbow joint angle data and averaged over these 8 repetitions.

#### Statistical analysis

Descriptive data are presented as means ± SDs. Two-way repeated ANOVA (2 conditions × 2 exercise types) was used to test the effects of condition (sling and ground) and exercise type (static and dynamic) and their interaction on % EMGmax value for each muscle. When a significant interaction was found, paired Student’s t-test was used to test the differences in the % EMGmax values between sling and ground conditions for each of static and dynamic exercises, but the comparisons between static and dynamic exercises were not performed because it was not the purpose of this study. Statistical significance was set at *P* < 0.05. As indices of effect size, *r* (for t-tests) and partial *η*^2^ (for ANOVA) values were reported with *P* values. All data were analyzed using SPSS software (SPSS Statistics 20; IBM, Japan).

### Results

**Figure 4:**
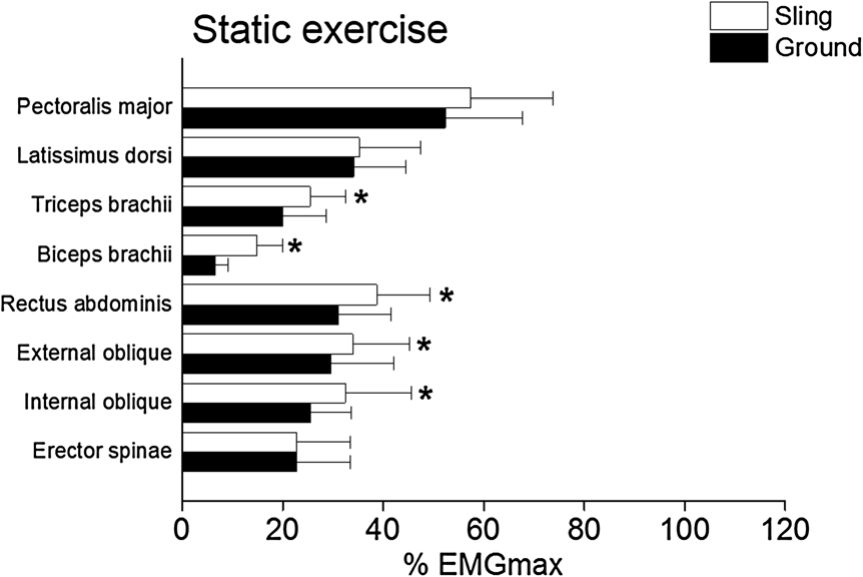
**Muscular activity levels during the static push-up exercise in the sling (open bar) and the ground (closed bar) conditions.** Values are means ± SDs. * indicates that % EMGmax in the sling condition is significantly higher (*P* < 0.05) than that in the ground condition.

**Figure 5:**
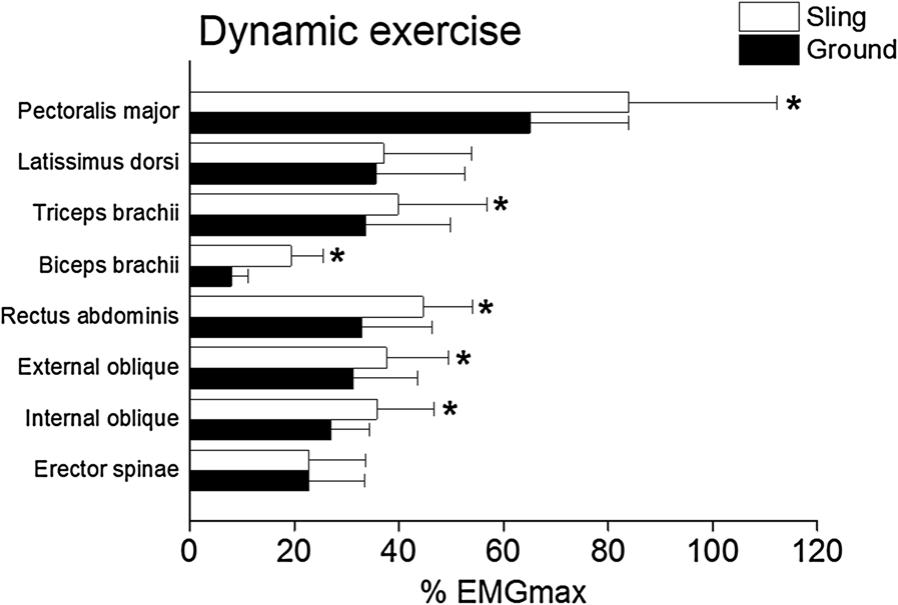
**Muscular activity levels during the dynamic push-up exercise in the sling (open bar) and the ground (closed bar) conditions.** Values are means ± SDs. * indicates that % EMGmax in the sling condition is significantly higher (*P* < 0.05) than that in the ground condition.

Figures 4 and 5 shows the muscular activity levels during static and dynamic push-up exercises performed under sling and ground conditions. In the pectoralis major muscle, there was a significant interaction between condition and type of exercise (*P* = 0.006, partial *η*^2^ = 0.336). In static exercise, there was no difference in % EMGmax values in sling and ground conditions (sling: 57.4 ± 16.4% vs. ground: 52.4 ± 15.3%). In dynamic exercise, sling condition showed significantly (*P* < 0.001, *r* = 0.779) higher % EMGmax value than the ground condition (83.9 ± 28.4% vs. 65.0 ± 18.9%). In the latissimus dorsi muscle, there were neither significant main effects of condition and type of exercise, nor interaction between them. In the triceps brachii muscle, there were significant main effects of condition (*P* < 0.001, partial *η*^2^ = 0.721) and exercise type (*P* < 0.001, partial *η*^2^ = 0.592) without interaction between them, indicating that % EMGmax value in sling condition was significantly higher than that in ground condition in both static (25.5 ± 7.1% vs. 20.0 ± 8.6%) and dynamic (39.8 ± 17.0% vs. 33.6 ± 16.3%) exercise. In the biceps brachii muscle, there was a significant interaction between condition and type of exercise (*P* = 0.035, partial *η*^2^ = 0.214). In both static (14.9 ± 5.1% vs. 6.5 ± 2.6%, *P* < 0.001, *r* = 0.829) and dynamic exercise (19.4 ± 6.2% vs. 8.0 ± 3.2%, *P* < 0.001, *r* = 0.868), sling condition showed significantly higher % EMGmax value than the ground condition. In the rectus abdominis muscle, there were significant main effects of condition (*P* < 0.001, partial *η*^2^ = 0.567) and exercise type (*P* = 0.045, partial *η*^2^ = 0.201) without interaction between them, indicating that % EMGmax value in sling condition was significantly higher than that in ground condition in both static (38.7 ± 10.6% vs. 31.1 ± 10.5%) and dynamic (44.6 ± 9.4% vs. 32.9 ± 13.3%) exercise. In the external oblique muscle, there were significant main effects of condition (*P* < 0.001, partial *η*^2^ = 0.654) and exercise type (*P* = 0.041, partial *η*^2^ = 0.203) without interaction between them, indicating that % EMGmax value in sling condition was significantly higher than that in ground condition in both static (34.0 ± 11.3% vs. 29.5 ± 12.6%) and dynamic (37.6 ± 11.8% vs. 31.2 ± 12.4%) exercise. In the internal oblique muscle, there were significant main effects of condition (*P* = 0.003, partial *η*^2^ = 0.378) and exercise type (*P* = 0.032, partial *η*^2^ = 0.220) without interaction between them, indicating that % EMGmax value in sling condition was significantly higher than that in ground condition in both static (32.4 ± 13.1% vs. 25.5 ± 8.0%) and dynamic (35.7 ± 10.9% vs. 27.0 ± 7.4%) exercise. In the erector spinae muscle, there were neither significant main effects of condition and type of exercise, nor interaction between them.

### Discussion

The main findings obtained here were that the sling-based push-up exercise showed significantly higher % EMGmax values than the ground-based push-up exercise in the upper limb and abdominal muscles in both static and dynamic exercises, and such condition-related difference in the pectoralis major was also significant in dynamic exercise. These results indicate that sling-based push-up exercise provides greater activation in both upper limb and anterior trunk muscles than the ground condition.

In both static and dynamic exercises, abdominal muscles showed significantly higher % EMGmax values in the sling than in the ground condition (Figures 4 and 5). As mentioned earlier, increased abdominal muscle activation when performing a push-up exercise under sling-based compared to ground-based condition has been reported [10,16]. It is considered that the demands of an unstable surface cause an increase in trunk muscle activation in order to maintain postural equilibrium during a given exercise or to complete exercise in a controlled manner [20,21]. Thus, the current result agrees with the previous finding [10,16] concerning increased activation of abdominal muscles when performing push-ups under unstable condition. Also, upper limb (triceps and biceps brachii) muscles showed significantly higher % EMGmax values in the sling than in the ground condition for both static and dynamic exercises (Figures 4 and 5). Previous studies [22,23] which used other instability devices reported that performing exercise in an unstable condition increased the co-contraction of limb muscles to control the position of the limb and/or perform the task accurately [2,20]. The current result supports this theory, and increased co-contraction of upper limbs can be considered as the task-specificity of sling-based push-up exercise where unstable grips must be maintained in position by co-contraction of the elbow flexors and extensors. These results suggest that during static push-up exercise, upper limb and abdominal muscles mainly contribute to maintaining balance under the condition of sling-induced instability.

In dynamic exercise, % EMGmax value of the pectoralis major muscle was also significantly higher in the sling than in the ground condition (Figure 5). This indicates that sling-based push-up exercise can provide greater training stimulus not only to abdominal and/or upper limb muscles, but also to one of the most important agonist muscles for performing push-ups (i.e. pectoralis major). At the same time, this result also suggests that whether sling-based compared to ground-based push-up exercise induces greater muscle activity depends on a type of exercise performed because the condition-related difference in muscle activation of the pectoralis major was not significant in the static push-up exercise. Previous studies [24,25] have reported that the activities of trunk muscles during instability exercises increase as the difficulty of the task increases. Freeman et al. [16] reported that more dynamic push-ups induced more muscle activation and higher spine load than less dynamic ones. Based on these and the current results, it may be considered that the contribution of pectoralis major muscle to controlling the body movements during dynamic push-up exercise is larger than during static push-up exercise, and thus the sling-induced higher activity was apparent only in dynamic exercise. Although the electromyographic comparison between dynamic and static contractions is very complicated [26], our data are based on the comparison between those in the same contraction type (i.e. comparison between sling and ground conditions in each of the static and dynamic exercises). Thus, the current results indicate that sling-based push-up does not induce higher activity in the pectoralis major muscle during static exercise, but it does so during dynamic exercise.

As mentinoned earlier, sling exericses are now performed by a wide range of population, including patiants with lower back pain or in- and experienced individuals. All participants in this study were relatively fit male university students majoring in physical education, so the current results may not be generalizable to the majority of the population. However, the findings obtained here should assist those in selecting optimal exercise progresssion that involve both sling- and ground-based static and dynamic push-up exercises.

### Conclusion

The sling-based push-up exercise showed significantly higher % EMGmax values than the ground-based push-up exercise in the upper limb and abdominal muscles in both static and dynamic exercises, and such condition-related difference in the pectoralis major was also significant in dynamic exercise. These results indicate that as compared to ground-based push-up exercise, sling-based push-up exercise can provide greater activation in both upper limb and anterior trunk muscles.

## Competing interests

The authors declare that they have no competing interests.

## Authors’ contributions

SM and TC contributed to the conception and the design of the study, performed the acquisition and analysis of the data, and drafted the article. MY participated in the design of the study and contributed to the interpretation of the data. HK made contribution to the conception of the study, participated in the design and coordination, helped to draft the manuscript, and gave final approval of the version to be published. All authors read and approved the final manuscript.
